# Explainable artificial intelligence (XAI) for predicting the need for intubation in methanol-poisoned patients: a study comparing deep and machine learning models

**DOI:** 10.1038/s41598-024-66481-4

**Published:** 2024-07-08

**Authors:** Khadijeh Moulaei, Mohammad Reza Afrash, Mohammad Parvin, Shahin Shadnia, Mitra Rahimi, Babak Mostafazadeh, Peyman Erfan Talab Evini, Babak Sabet, Seyed Mohammad Vahabi, Amirali Soheili, Mobin Fathy, Arya Kazemi, Sina Khani, Seyed Mohammad Mortazavi, Sayed Masoud Hosseini

**Affiliations:** 1 Department of Health Information Technology, School of Paramedical, Ilam University of Medical Sciences, Ilam, Iran; 2 Deparment of Artificial Intelligence, Smart University of Medical Sciences, Tehran, Iran; 3 Department of Industrial and Systems Engineering, Auburn University, Auburn, AL USA; 4 Toxicological Research Center, Excellence Center of Clinical Toxicology, Department of Clinical Toxicology, Loghman Hakim Hospital, Shahid Beheshti University of Medical Sciences, Tehran, Iran; 5 Department of Surgery, Faculty of Medicine, Shahid Beheshti University of Medical Sciences, Tehran, Iran; 6 School of Medicine, Tehran University of Medical Sciences, Tehran, Iran; 7 Students Research Committee, School of Medicine, Shahid Beheshti University of Medical Sciences, Tehran, Iran; 8 Rajaie Cardiovascular Medical and Research Center, Iran University of Medical Sciences, Tehran, Iran

**Keywords:** Explainable artificial intelligence (XAI), Predict, Intubation, Methanol, Deep learning, Machine learning, Biotechnology, Engineering

## Abstract

The need for intubation in methanol-poisoned patients, if not predicted in time, can lead to irreparable complications and even death. Artificial intelligence (AI) techniques like machine learning (ML) and deep learning (DL) greatly aid in accurately predicting intubation needs for methanol-poisoned patients. So, our study aims to assess Explainable Artificial Intelligence (XAI) for predicting intubation necessity in methanol-poisoned patients, comparing deep learning and machine learning models. This study analyzed a dataset of 897 patient records from Loghman Hakim Hospital in Tehran, Iran, encompassing cases of methanol poisoning, including those requiring intubation (202 cases) and those not requiring it (695 cases). Eight established ML (SVM, XGB, DT, RF) and DL (DNN, FNN, LSTM, CNN) models were used. Techniques such as tenfold cross-validation and hyperparameter tuning were applied to prevent overfitting. The study also focused on interpretability through SHAP and LIME methods. Model performance was evaluated based on accuracy, specificity, sensitivity, F1-score, and ROC curve metrics. Among DL models, LSTM showed superior performance in accuracy (94.0%), sensitivity (99.0%), specificity (94.0%), and F1-score (97.0%). CNN led in ROC with 78.0%. For ML models, RF excelled in accuracy (97.0%) and specificity (100%), followed by XGB with sensitivity (99.37%), F1-score (98.27%), and ROC (96.08%). Overall, RF and XGB outperformed other models, with accuracy (97.0%) and specificity (100%) for RF, and sensitivity (99.37%), F1-score (98.27%), and ROC (96.08%) for XGB. ML models surpassed DL models across all metrics, with accuracies from 93.0% to 97.0% for DL and 93.0% to 99.0% for ML. Sensitivities ranged from 98.0% to 99.37% for DL and 93.0% to 99.0% for ML. DL models achieved specificities from 78.0% to 94.0%, while ML models ranged from 93.0% to 100%. F1-scores for DL were between 93.0% and 97.0%, and for ML between 96.0% and 98.27%. DL models scored ROC between 68.0% and 78.0%, while ML models ranged from 84.0% to 96.08%. Key features for predicting intubation necessity include GCS at admission, ICU admission, age, longer folic acid therapy duration, elevated BUN and AST levels, VBG_HCO3 at initial record, and hemodialysis presence. This study as the showcases XAI's effectiveness in predicting intubation necessity in methanol-poisoned patients. ML models, particularly RF and XGB, outperform DL counterparts, underscoring their potential for clinical decision-making.

## Introduction

Methanol (CH3OH) is a hazardous alcohol commonly present in household and industrial products. Exposure to methanol poses grave risks, leading to substantial morbidity and mortality if not promptly addressed. Methanol poisoning typically results from accidental or intentional ingestion, with accidental outbreaks occasionally occurring due to errors in distillation, fermentation, or beverage contamination. Exposure to methanol can result in varying degrees of toxicity, necessitating a spectrum of treatments ranging from close laboratory monitoring to antidotal therapy and dialysis^1^. Methanol often induces hypotension through vasodilation and vomiting, necessitating intravenous crystalloid hydration for many patients. Additionally, methanol exposure can lead to coma and respiratory arrest. So, mandating intubation and mechanical ventilation for individuals experiencing severe poisoning^2^. Intubation is a life-saving intervention in cases of severe respiratory failure, unmatched in efficacy by most medical therapies^3^.

The requirement for intubation in acutely ill patients is often urgent and unpredictable for physicians^4^. The need for intubation in methanol-poisoned patients typically follows clinical guidelines and protocols based on the severity of the poisoning and the patient's symptoms. These standards generally consider factors such as the level of consciousness, respiratory distress, metabolic acidosis, hypotension by vasodilation, vomiting, and the presence of visual disturbances^2^. Therefore, accurately predicting the necessity for intubation at an early stage can offer additional time for preparation, thereby enhancing safety margins and preventing the potential risks associated with delayed intubation^5^. Recognizing patients experiencing progressive respiratory decline sooner could transform an urgent need for intubation into a planned, elective procedure, ultimately reducing certain related health complications^6^. Additionally, repeated intubation attempts are linked to a heightened risk of complications including cardiac arrest, hypoxemia, arrhythmia, regurgitation, and airway trauma^4^. Various Artificial intelligence (AI) techniques such as machine learning (ML) and deep learning (DL) can accurately predict the need for intubation in people and prevent many unwanted complications and even death^5,7,8^.

The findings from the study by Im et al.^7^, demonstrate that utilizing a multimodal deep neural network (MDNN) model integrating clinical data and time-series variables accurately predicted the need for intubation within the next 3 h in neonates with respiratory distress, achieving an accuracy of 88.2%. This suggested model could assist in guiding decision-making for neonates experiencing respiratory distress necessitating endotracheal intubation^7^. Additonally, the findings from the study conducted by Siu et al. demonstrated that machine learning techniques could forecast the requirement for intubation in critically ill patients by leveraging routinely gathered bedside clinical parameters and laboratory findings. This tool could potentially be deployed in real-time to aid clinicians in predicting the necessity for intubation within 24 h of admission to the intensive care unit^5^. Other study also show that algorithms have the potential to enhance conventional clinical criteria for predicting the need for intubation in hospitalized patients. These ML-based prediction models could assist physicians in optimizing the timing of intubation, facilitating better allocation of mechanical ventilation resources and personnel, and improving patient clinical outcomes^8^.

To our knowledge, no study has utilized DL and ML techniques to predict the need for intubation in methanol-poisoned patients. The studies conducted cover predicting intubation needs in various contexts: among COVID-19 patients^8^, in the emergency department^4^, within 3 h in neonatal intensive care units using a multimodal deep neural network^7^, and within the first 24 h after critical care admission using machine learning approaches^5^. In these studies, only ML or DL techniques have been used; these two techniques have not been compared, and they have not focused on the need for intubation in methanol-poisoned patients. The aim of the present study is to compare deep learning and machine learning models for predicting the need for intubation in methanol-poisoned patients (Supplementary information).

## Methods

### Study design and setting

This retrospective observational study was conducted in 2024 using the Transparent Reporting of a Multivariable Prediction Model for Individual Prognosis or Diagnosis (TRIPOD) guideline (Appendix A). It utilized a dataset comprising 897 patients poisoned with methanol, including records of both patients needing intubation and those who did not, from Loghman Hakim Hospital in Iran, Tehran. The primary objective of this study was to examine the necessity for intubation in methanol-poisoned patients. To predict the need for intubation in methanol-poisoned patients, eight established ML and DL models were deployed. These models leveraged an array of clinical and demographic features from the dataset to make accurate predictions. To mitigate the risk of overfitting, the training of these models incorporated a robust tenfold cross-validation approach, ensuring their generalizability and reliability.

### Data set description and participants

Dataset involves methanol-poisoned patients requiring intubation and covers admissions from March 17, 2020, to March 20, 2024. The dataset comprises 897 records of patients poisoned with methanol from Loghman Hakim Hospital. This hospital acts as the primary destination for referrals for individuals affected by poisoning. Within this dataset, there were 202 cases of methanol-poisoned patients requiring intubation and 695 cases of methanol-poisoned patients who did not require intubation. The confirmation of methanol poisoning involved reviewing medical records for evidence of methanol exposure, serum methanol levels surpassing 6.25 mmol/L (20 mg/dL), or the manifestation of clinical symptoms such as visual disturbances, abdominal pain, breathing difficulties, and neurological symptoms, alongside a pH level below 7.3 and serum bicarbonate levels below 20 mmol/L upon admission.

**Figure 1:**
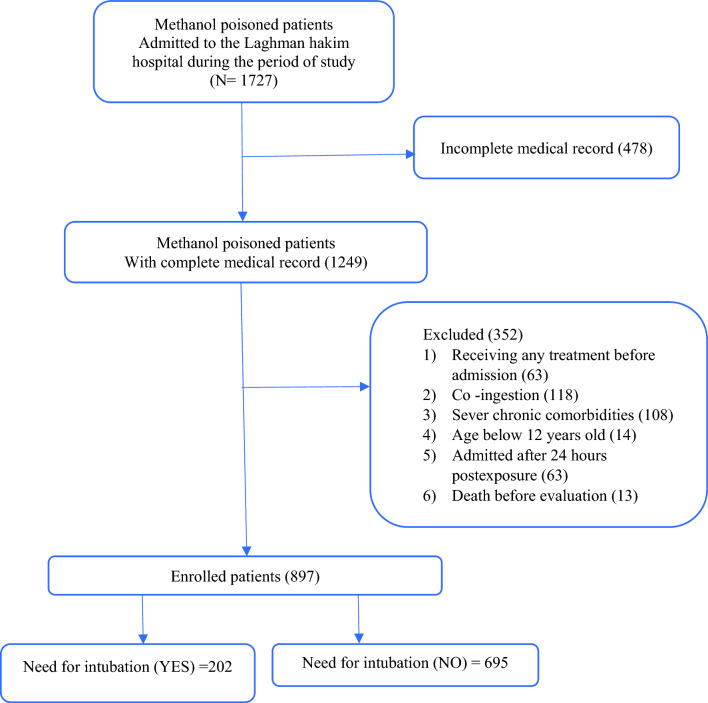
Patients’ selection flowchart.

The patient selection process is depicted in Fig. 1. The study included individuals aged 12 and above who were hospitalized within 24 h of confirmed methanol poisoning. The criteria for exclusion included the simultaneous ingestion of substances besides ethanol, the administration of any pre-admission therapies that might influence the analysis, severe chronic conditions (such as cardiovascular disease, chronic kidney disease, chronic liver disease, diabetes, chronic obstructive pulmonary disease, blood disorders, malignancy, etc.), mortality before assessment, and incomplete medical documentation. Patients were categorized into two groups: those necessitating intubation and those not requiring it.

It should be noted that there is no special classification for intubating patients poisoned with methanol. Typically, these patients are intubated based on clinical symptoms, laboratory findings (such as blood methanol levels and metabolic acidosis), and overall health status. Consequently, in this study, only the patients who met the established clinical criteria for intubation were included.

### Data collection

Six individual researchers conducted a thorough review of the patients' medical records. The original questionnaire, obtained from the electronic databases of Loghman Hakim Hospital (Sabara and Shafa databases), was utilized to gather clinical data.

This questionnaire encompassed details regarding age, gender, vital signs (including respiratory rate, blood pressure, body temperature, and pulse rate), medical history (including underlying conditions), mental status (including agitation, confusion, seizures, and GCS score), visual symptoms upon admission, ingested dose, antidote therapy, and laboratory test results (including hemoglobin, platelet count, white cell count, serum creatinine (sCr), blood glucose, alanine transaminase (ALT), creatine phosphokinase (CPK), aspartate transaminase (AST), sodium, potassium, alkaline phosphatase (ALP), venous blood gas analysis (pH, PCO2, and HCO3), and blood urea nitrogen (BUN)). Furthermore, hospital-related factors such as the requirement for intubation and duration of hospitalization were recorded.

### Pre-processing of the data

To prepare the data for analysis, we began by importing the dataset into Jupyter Notebook within the Anaconda environment, utilizing Python version 13.1. For effective data preprocessing, we relied on the NumPy library, essential for handling array operations and mathematical functions. NumPy facilitates various preprocessing tasks, such as managing missing values and conducting statistical calculations, which streamline data manipulation and pave the way for deeper analysis. Initially, missing values of certain variables were replaced with mean and mode statistical measures. Subsequently, nominal values of variables in the columns were converted to numerical values for improved results, employing variable encoding to enhance algorithm learning. Finally, incomplete rows of data (with missing values exceeding 70%) were removed. This systematic approach ensured that our dataset was clean and ready for analysis, minimizing the potential for biased results and enhancing the reliability of our findings.

Afterwards, we applied Min–max normalization to the data. This involved identifying and removing outliers from the dataset to enhance its quality. Min–max normalization adjusts numerical data to a predetermined range, usually 0 and 1, while preserving the relative relationships between values. This method is employed in data normalization to foster uniform and standardized feature scales, preventing certain features from dominating others during analysis. By preserving proportional relationships between values, Min–max normalization guarantees equitable comparison and precise interpretation of the dataset across various features.

### Feature selection

The main purpose of feature selection in machine learning is to pinpoint the best features or key parameters to enhance model performance. Among 187 evaluated features, 110 were excluded due to incomplete medical records and missing data. The remaining 77 features underwent Pearson’s correlation coefficient analysis, resulting in the identification of 43 significant features for predicting methanol poisoning prognosis. Features with near-zero correlation and linear data representation were removed. These 43 features were then integrated into machine learning models. Afterwards, the FeatureWiz library was utilized for another round of feature selection, leading to 23 selected features. Feature selection using the FeatureWiz library involves two main stages. Initially, the Searching for the Uncorrelated List of Variables (SULOV) method identifies variable pairs outside the correlation threshold. Subsequently, the Mutual Information Score (MIS) of these pairs is calculated, and the pair with the lowest correlation and highest MIS is chosen for further analysis. In the next phase, variables selected through SULOV are iteratively processed through XGboost to identify optimal features based on the target variable, thus reducing the dataset size. This method helps in selecting the most impactful predictive features from the dataset.

### Data analysis software

In this study, we extensively employed the Python programming language (version 13.1) along with various associated libraries. We utilized Jupyter Notebook within the Anaconda environment, utilizing Python version 13.1. Matplotlib, NumPy, Seaborn, and Pandas were utilized for data analysis and visualization, while the scikit-learn library facilitated the development and evaluation of machine learning models Deep learning architectures, were constructed and trained using TensorFlow. Furthermore, model interpretability and feature importance analyses were conducted using SHAP (SHapley Additive exPlanations), and LIME (Local Interpretable Model-agnostic Explanations)."

### Machin learning and deep learning models development

In total, we utilized eight well-known models from both the deep learning (DL) and machine learning (ML) realms for prediction the need for intubation in methanol-poisoned patients. Among the DL models employed were the Deep Neural Network (DNN), feedforward neural network (FNN), Long Short-Term Memory (LSTM), and Convolutional Neural Network (CNN). Conversely, the ML models encompassed Extreme Gradient Boosting (XGB), Support Vector Machine (SVM), Decision Tree (DT), and an additional Random Forest (RF).

These selected models offer a diverse array of methodologies suitable for diseases prediction, encompassing both deep learning and machine learning approaches. Deep learning models like DNN, FNN, LSTM, and CNN excel in capturing intricate patterns within the data, whereas ML models such as XGB, SVM, DT, and RF provide robust and easily interpretable predictions. By leveraging this range of models, our objective was to enhance prediction accuracy and gain insights into the complex factors influencing the need for intubation in methanol-poisoned patients.

It should be noted that, while it is true that CNNs are predominantly used for image data due to their ability to capture spatial hierarchies, recent studies^9,10^ have demonstrated their potential in handling tabular data as well. CNNs can effectively learn local dependencies and patterns within tabular data, similar to how they detect features in images. The findings of Buturović et al.'s study^9^ showed that CNNs can perform accurately in predicting diseases using tabular data.

### Cross-validation and hyperparameter tuning

To mitigate the risk of overfitting, we incorporated tenfold cross-validation during the training of all proposed models. This approach entails partitioning the dataset into 10 equally sized folds, with the model trained on 9 folds and validated on the remaining fold in each iteration. This iterative process is repeated 10 times to ensure comprehensive validation. The ultimate performance metric is computed by averaging the outcomes from these iterations, offering a dependable evaluation of the model's effectiveness^11^.

The process of optimizing models for a specific dataset involves the careful selection and adjustment of hyperparameters to create the most effective model. The selection of hyperparameters plays a crucial role in determining the overall performance of a specific machine learning algorithm. After completing the preprocessing phase, a sequence of machine learning (ML) and deep learning (DL) modeling tasks were initiated to fine-tune and optimize these hyperparameters. This iterative approach was geared towards pinpointing the ideal hyperparameter configurations necessary for developing models with the highest F-score. In this investigation, we employed the GridSearchCV technique to pinpoint the most precise and resilient models. The hyperparameters of the optimal model, the Gradient Boosting Classifier, were adjusted as follows: (learning_rate = 0.2, max_depth = 5, n_estimators = 10, min_samples_leaf = 30, subsample = 0.8, min_samples_split = 400, random_state = 10, max_features = 9)^12^.

### Explanation and justification the output of ML and DL models

ML and DL methods are often regarded as "black box" models due to their intricate inner workings, posing challenges for interpretation^13,14^.This lack of interpretability can be particularly problematic in critical fields like healthcare, where understanding prediction rationales is vital. To tackle this issue, researchers have focused on enhancing model interpretability. Two notable techniques are Shapley Additive Explanations (SHAP) and Local Interpretable Model-agnostic Explanations (LIME), which offer insights into ML model predictions^15,16^. In our study, both SHAP and LIME were utilized as interpretability methods in machine learning. While both methods serve the purpose of explaining model predictions, they have distinct characteristics and can provide complementary insights.

SHAP, drawing from Shapley values in cooperative game theory, has garnered attention across various fields, including clinical studies^17,18^. It assigns contribution values to dataset features, showing their impact on predicted outcomes. These values are derived by comparing predictions with and without specific features. Through examining all feature combinations, SHAP provides a holistic understanding of each feature's contribution, aiding researchers in identifying their impact on outcomes^17^. Moreover, SHAP offers a theoretical framework rooted in cooperative game theory, providing globally consistent explanations by assigning each feature an importance value based on its contribution to the model's output. This method offers a comprehensive understanding of feature importance across the entire dataset^19^.

LIME is an algorithm aimed at clarifying predictions made by any classifier or regressor by creating a local interpretable model. It prioritizes interpretability and local fidelity, facilitating a qualitative understanding of the input–output relationship and ensuring the model's reliability near the predicted instance. As a model-agnostic tool, LIME can elucidate any model's predictions, treating it as a black box. It demonstrates versatility by interpreting image classifications, providing insights into text-based models, and explaining tabular datasets in various formats textual, numeric, or visual^16^.

In essence, SHAP and LIME are invaluable for interpreting ML and DL model predictions, boosting transparency and trust in decision-making processes. Their use in healthcare settings aids clinicians in understanding and validating AI predictions, facilitating informed decisions^16,20^. In this study, SHAP and LIME shed light on feature influences on predicted outcomes for both ML and DL models. Consequently, SHAP and LIME diagrams were created for the top-performing model across sensitivity, specificity, accuracy, ROC, and F1-score indices.

Therefore, by employing both SHAP and LIME, we aimed to leverage the strengths of each method to obtain a more holistic understanding of our model's behavior. While one method may suffice in certain scenarios, the combined use of SHAP and LIME allowed us to validate and cross-reference the interpretability of our model across different scales and contexts.

### Performance evaluation of models

The ML and DL models' performance underwent a thorough evaluation utilizing performance metrics obtained from the confusion matrix, as detailed in Table 1. The assessment of predictive models encompassed a range of essential metrics including accuracy, specificity, sensitivity, F1-score, and the receiver operating characteristic (ROC) curve, all presented in Table 2.

**Table 1:** Confusion matrix.

Output		Prediction value	
		Death ( +)	Living
Real value	Death ( +)	TP	FN
	Living ( −)	FP	TN

*True positive (TP): The number of deaths that the model has correctly identified.

*False positive (FP): The number of living people but the model has incorrectly identified them as dead.

*True negative (TN): The number of people who are living and the model correctly identified them as living.

*False negative (FN): The number of people who are dead but the model has identified them as living incorrectly.

**Table 2:** The performance evaluation measures.

Evaluation measures	Formula
Accuracy	(TP + TN)/ (TP + FP + TN + FN)
Sensitivity/ Recall	TP/ (TP + FN)
Specificity	TN/ (TN + FP)
F1-measure	2* ((Precision * Sensitivity) / (Precision + Sensitivity))

### Ethical considerations

The study received approval from the ethics committee of Shahid Beheshti University of Medical Sciences, identified by reference number IR.SBMU.RETECH.REC.1402.826. All methods were performed in accordance with the relevant guidelines and regulations by ethics committee of Shahid Beheshti University of Medical Sciences. In cases where participants were unable to provide consent themselves, consent was obtained from participants or their families. The informed consent obtained at our institutions also included authorization for potential future retrospective analyses.

## Results

Table 3 shows the features of patients included in the study. The research involved 897 participants, with an average age of 33.41 years and a Std of 13.93, spanning an age range from 13 to 82 years. Among the participants, 724 were male, averaging 34.55 ± 14.46 years old, and 173 were female, averaging 28.64 ± 10.178 years old (Table 3).

**Table 3:** Features of patients.

Features	Count	Value	Frequency	Mean	Std
Age	897		897	33.41	13.94
Sex	897	Men	724	34.55	14.46
		Women	173	28.64	10.178
Co_ingestion	897	Co_ingestion	897	–	–
Ingested_dose	897	Ingested_dose	897	–	–
Alcohol_addiction	897	Yes	80	–	–
		No	817		
Candidate_for_dialysis_at_admission	897	Yes	108	–	–
		No	789		
Underlying_disease	897	Yes	39	–	–
		No	858		
Visual_symptom_at_admission	897	Yes	197	–	–
		No	700		
Respiratory_symptom_at_admission	897	Yes	140	–	–
		No	757		
Confusion_at_admission	897	Yes	194	–	–
		No	703		
Agitation_at_admission	897	Yes	109	–	–
		No	788		
Seizure_at_admission	897	Yes	28	–	–
		No	869		
Hemodialysis	897	Yes	Yes	–	–
		No	No		
Antidote_therapy	897	Yes	Yes	–	–
		No	No		
ICU_admission	897	Yes	160		
		No	599		
Intubation	897	Yes	202		
		No	695		
Ingestion_admission_Time_span_h	897			32.50	19.11
Kind_of_visual_symptom_at_admission	897			0.926	0.78
Ethanol_therapy_dosage	897			70.83	58.65
Ethanol_therapy_duration	897			9.77	12.95
Folic_acid_therapy_dosage	897			49.377	43.47
Folic_acid_therapy_duration	897			11.30	13.67
T_at_admission	897			36.89	0.34
PR_at_admission	897			87.38	12.31
RR_at_admission	897			16.51	3.78
DBP_at_admission	897			76.85	11.75
SBP_at_admission	897			120.51	17.27
GCS_at_admission	897			13.19	3.31
VBG_PH_First_record	897			7.19	0.22
VBG_PCO2_First_record	897			31.36	12.9
VBG_HCO3_First_record	897			15.84	7.42
K_First_record	897			4.34	0.68
Na_First_record	897			138.97	5.67
BUN_First_record	897			17.81	11.22
CPK_First_record	897			270.35	392.17
WBC_First_record	897			10.97	5.74
PLT_First_record	897			258.61	82.86
Hb_First_record	897			16.4	2.34
Cr_First_record	897			1.27	0.33
BS_First_record	897			140.62	74.12
AST_First_record	897			46.77	50.4
ALT_First_record	897			49.3	189.06
ALP_First_record	897			235.9	96.2

### Optimal parameters using hyperparameter tuning

Table 4 exhibits various models alongside their parameters, demonstrating the optimal parameters obtained through hyperparameter tuning and majority voting.

**Table 4:** Optimal parameters using hyperparameter tuning.

Num	ML & DP models	Hyper-Parameters
1	CNN	Conv1D(filters = 32, kernel_size = 3, MaxPoolingD (max pool_size = 2), Dense (units = 16)
2	LSTM	LSTM Units = 64, Activation Function = 'sigmoid', Optimizer = 'adam', Loss Function = 'binary_crossentropy', Epochs = 50, Batch Size = 32
3	DNN	Layers and Neurons = 128,64,1 Activation Functions = 'relu', 'relu', 'sigmoid',Optimizer = 'adam', Loss Function = 'binary_crossentropy', Epochs = 40, Batch Size = 64
4	FNN	Hidden Layer Size = (128,), Max Iterations = 2000, Random State = 42, Activation Function = 'relu' , Solver = 'adam', Learning Rate = 'constant', Batch Size = '128'
5	RF	n_estimators = 100,criterion = ' gini, max_depth = 8, min_samples_split = 2, min_samples_leaf = 1, max_features = 'auto', bootstrap = True, oob_score = False, random_state = 42
6	DT	Criterion = 'gini', max_depth = 8, min_samples_split = 10, min_samples_leaf = 5, max_features = 'sqrt', splitter = 'best', random_state = 42
7	XGB	learning_rate = 0.03, max_depth = 4, n_estimators = 150, min_child_weight = 3, colsample_bytree = 0.8, subsample = 0.7, gamma = 0.2, reg_lambda = 1.5
8	SVM	Probability = ’True’, C = ’ 100.0’, kernel = 'linear' , degree = ’ 1 ‘ gamma = scale

### Performance evaluation of selected models

Table 5 displays the performance evaluation results of ML and DL models. Among all DL models, LSTM performed better in terms of accuracy (94.0%), sensitivity (99.0%), specificity (94.0%), and F1-score (97.0%). Additionally, among these algorithms, CNN had the highest ROC (78.0%).

**Table 5:** Performance evaluation of selected models.

Models	Accuracy (%)	Sensitivity (%)	Specificity (%)	F1-score (%)	ROC (%)
Performance of each deep learning models					
CNN	92.0	96.6	92.0	96.6	**78.0**
LSTM	**94.0**	**99.0**	**94.0**	**97.0**	74.0
DNN	78.0	93.0	78.0	93.0	74.0
FNN	78.0	93.0	78.0	93.0	68.8
Performance of each machine learning models					
RF	**97.0**	98.0	**100**	98.0	85.0
DT	93.0	96.0	93.0	96.0	85.0
XGB	96.88	**99.37**	96.88	**98.27**	**96.08**
SVM	96.00	99.00	96.0	98.0	84.0

Significant values are in bold.

Among all ML models, RF performed better in terms of accuracy (97.0%) and specificity (100%). Following the RF model, XGB performed better in terms of sensitivity (99.37%), F1-score (98.27%), and ROC (96.08%).

In general, the performance of RF, with accuracy (97.0%) and specificity (100%), and XGB, with sensitivity (99.37%), F1-score (98.27%), and ROC (96.08%), was superior to that of all other models. Additionally, the performance of ML models was better than that of DL models in all performance measures. DL models (CNN, LSTM, DNN, and FNN) demonstrated accuracies ranging from 78.0% to 94.0%, whereas ML models (RF, DT, XGB, and SVM) attained accuracies between 93.0% and 97.0%. ML models exhibited sensitivities ranging from 93.0% to 99.0%, while DL models demonstrated sensitivities between 98.0% and 99.37%. DL models demonstrated specificities ranging from 78.0% to 94.0%, whereas ML models achieved specificities between 93.0% and 100%. In terms of F1-scores, DL models ranged from 93.0% to 97.0%, while ML models ranged between 96.0% and 98.27%. Moreover, DL models attained ROC scores between 68.0% and 78.0%, while ML models ranged from 84.0% to 96.08% in ROC scores.

**Figure 2:**
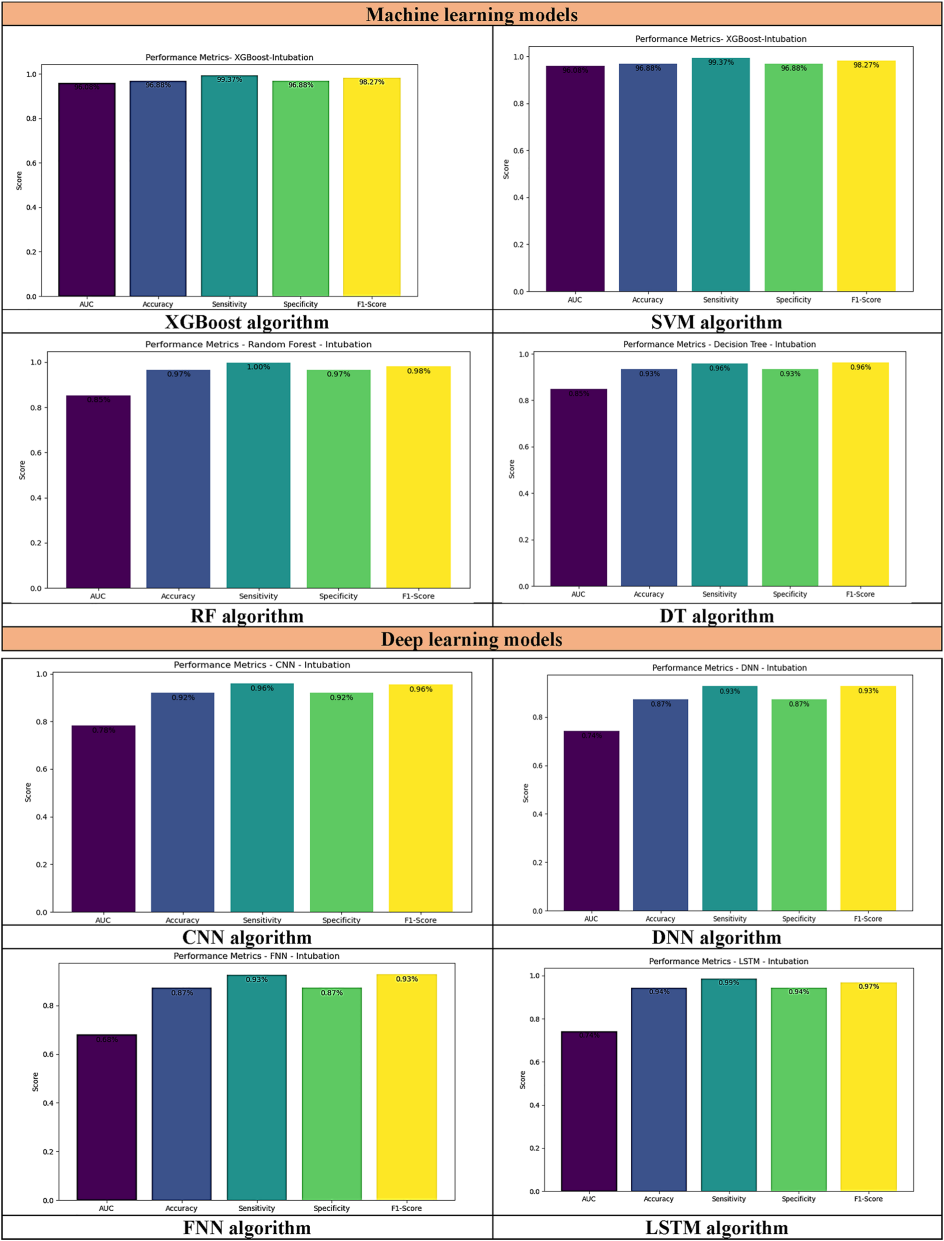
Performance evaluation of ML and DL models for predicting the need for intubation in methanol-poisoned patients.

**Figure 3:**
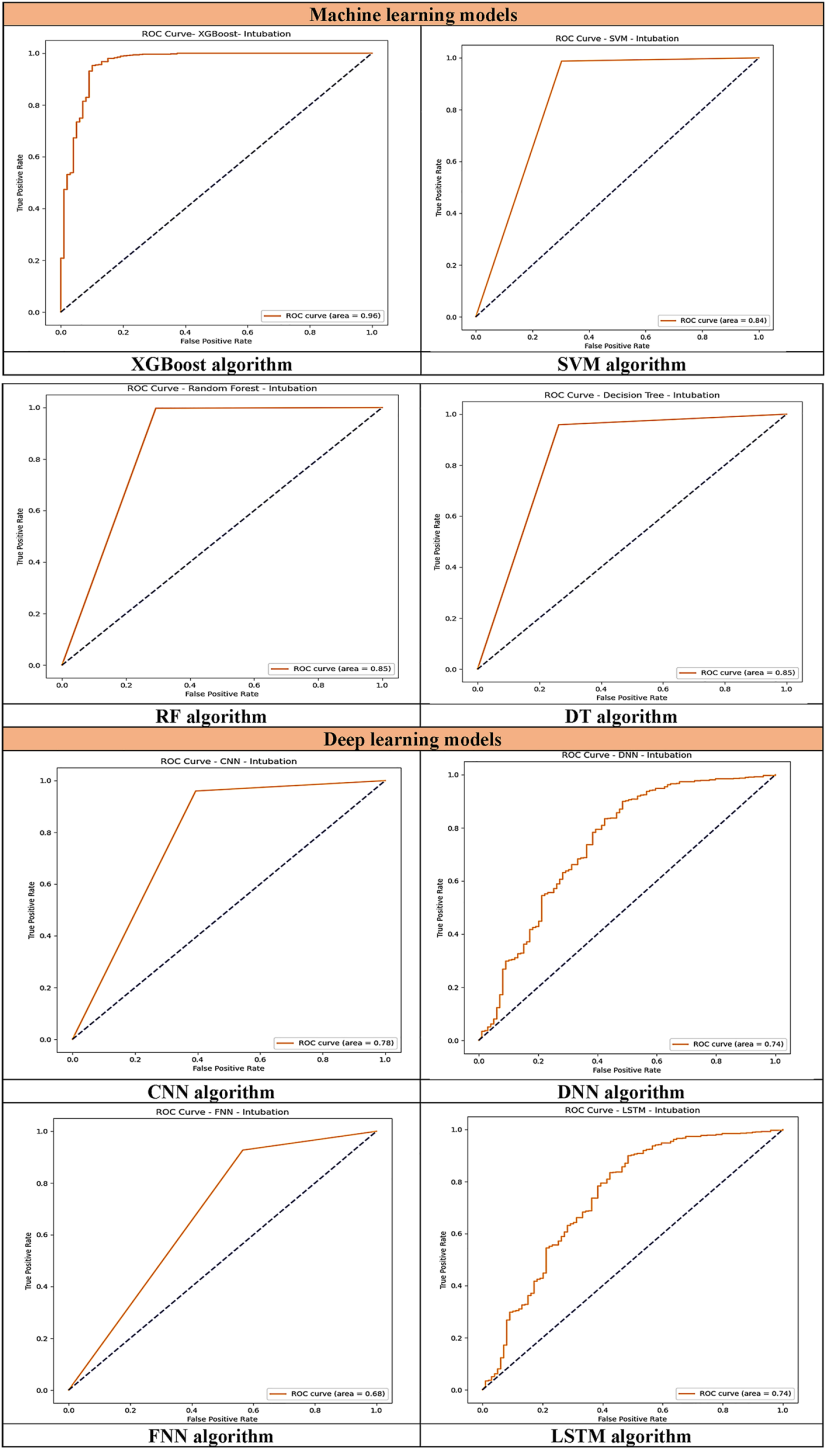
ROC curves comparing ML and DL models for predicting the need for intubation in methanol-poisoned patients.

Figure 2 depicts the performance of chosen ML and DL models in predicting the need for intubation in methanol-poisoned patients, while Fig. 3 compares their respective ROC curves.

## Explanation and justification the output of ML and DL models

### Shapley additive explanations (SHAP)

**Figure 4:**
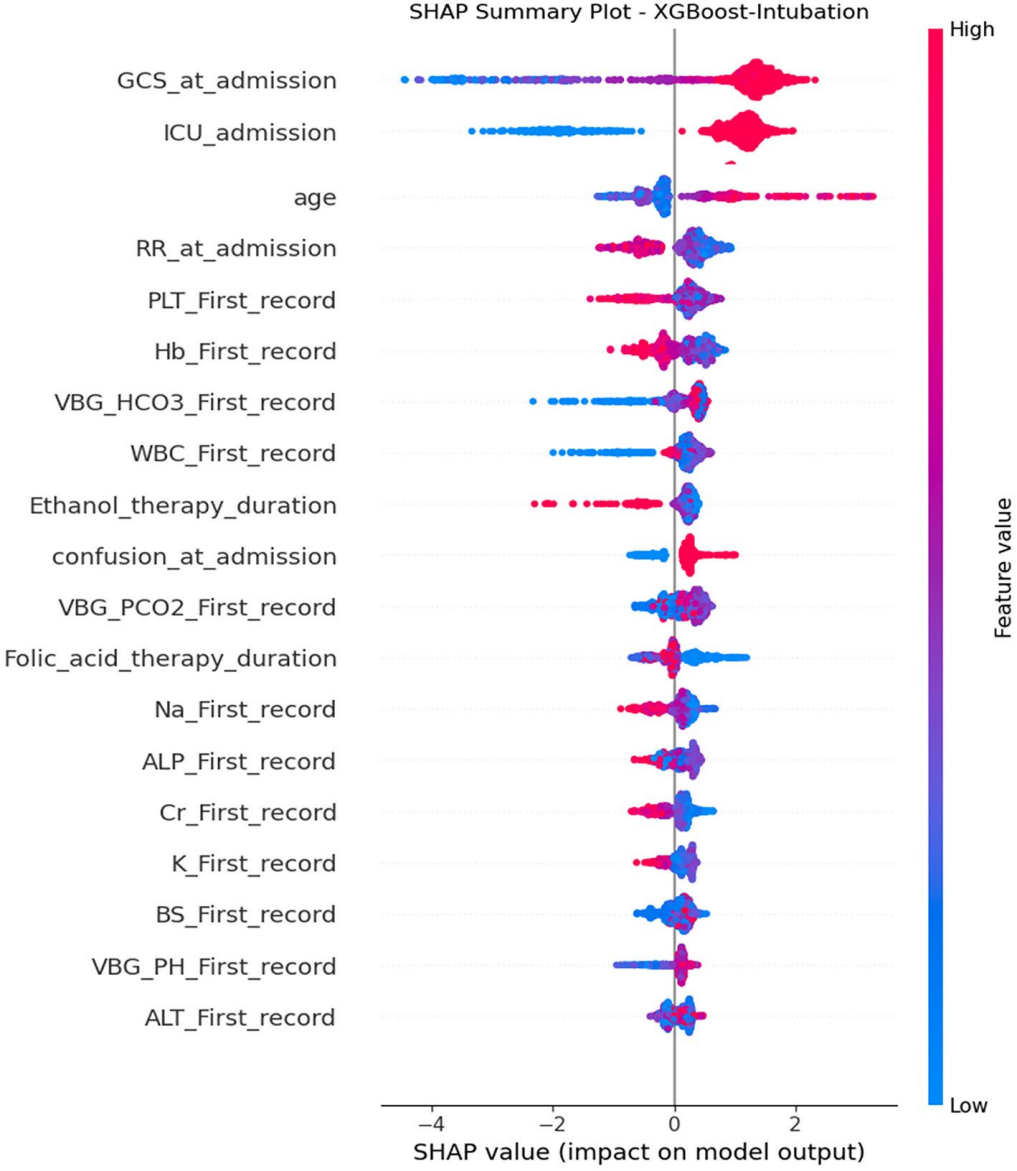
SHAP summary plot for XGB model.

As noted previously, the XGB algorithm outperformed all others in terms of sensitivity, specificity, and ROC. Consequently, a SHAP plot was specifically generated for this algorithm. Figure 4 shows the SHAP summary plot. The SHAP figure presents the hierarchy of feature importance in descending order along the y-axis, while the x-axis denotes the corresponding SHAP values. A negative SHAP value suggests a reverse association between a particular feature and the outcome, whereas positive values indicate a direct correlation. As can be seen in the figure below, GCS_at_admission, ICU_admission, Intubation-at-admission, and age were the most important features for predicting the need for intubation in methanol-poisoned patients.

### Local interpretable model-agnostic explanations (LIME)

**Figure 5:**
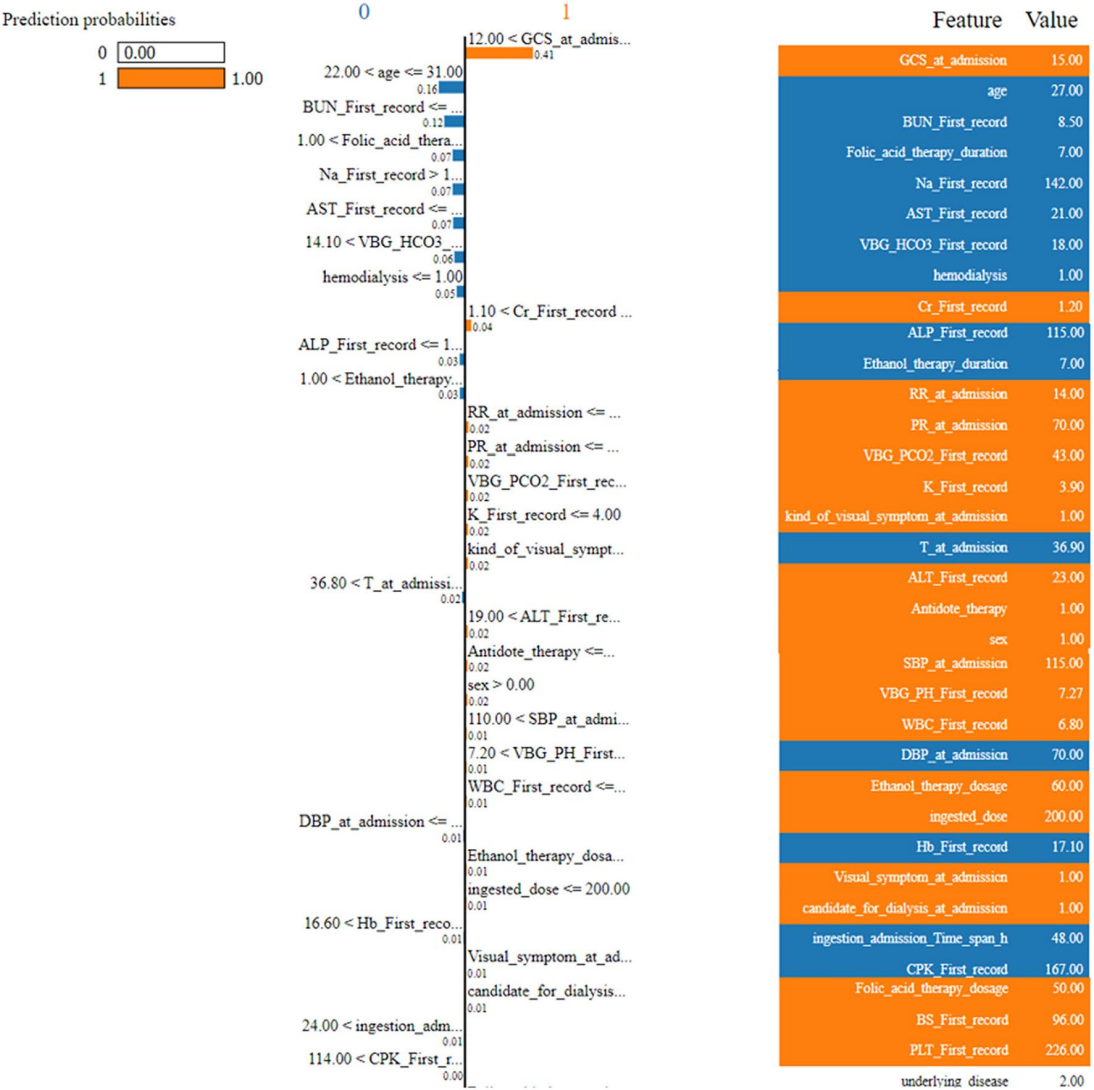
LIME summary plot for XGB model.

Figure 5 shows the LIME summary plot. The left panel shows the prediction probabilities, where the model assigns a probability of 1.0 to the positive class (presumably indicating the need for intubation). The middle panel displays the feature contributions, where longer bars to the right indicate features that contribute more towards the positive class prediction. Some notable features contributing positively to the need for ventilation include high GCS (Glasgow Coma Scale) at admission, younger age, longer duration of folic acid therapy, high levels of BUN (blood urea nitrogen), AST (aspartate aminotransferase), and VBG_HCO3 (venous blood gas bicarbonate) at first record, and the presence of hemodialysis.

On the other hand, features like high creatinine and ALT (alanine aminotransferase) levels at first record, high SBP (systolic blood pressure) at admission, and certain comorbidities like agitation, seizure, seem to contribute negatively towards the prediction.

The right panel shows the actual feature values for this specific case, providing context for interpreting the feature contributions.

## Discussion

In our study titled "Explainable Artificial Intelligence (XAI) for predicting the need for intubation in methanol-poisoned patients: A study comparing deep and machine learning models," we evaluated the performance of various DL and ML models in predicting intubation necessity. Our results, as the first study to predict the need for intubation in methanol-poisoned patients, revealed that among DL models, LSTM demonstrated superior accuracy, sensitivity, specificity, and F1-score, while CNN led in ROC. Conversely, RF and XGB excelled among ML models, with RF achieving high accuracy and specificity, and XGB exhibiting excellent sensitivity, F1-score, and ROC. Overall, RF and XGB outperformed other models across multiple metrics, indicating the superiority of ML over DL models in predicting intubation necessity. Additionally, we identified key features such as Glasgow Coma Scale (GCS) at admission, ICU admission, age, and biochemical markers as crucial predictors for intubation necessity in methanol-poisoned patients.

As mentioned above, our study findings indicated that LSTM and CNN exhibited superior performance. Specifically, among DL models, STM demonstrated superior accuracy, sensitivity, specificity, and F1-score. our study highlights the superior performance of LSTM and CNN models in predicting intubation necessity, each demonstrating distinct strengths. LSTM exhibited exceptional accuracy, sensitivity, specificity, and F1-score, suggesting its proficiency in capturing intricate patterns relevant to intubation in methanol-poisoned patients. Conversely, CNN excelled in ROC performance, efficiently capturing spatial hierarchies crucial for clinical decision-making. While LSTM's overall superiority is evident, CNN's ability to distinguish between intubation and non-intubation cases underscores its clinical relevance. Moreover, the effectiveness of DL models, particularly LSTM and Bi-LSTM, in predicting intubation for ICU patients, as demonstrated by Lee et al.^21^, and the outperformance of LSTM networks in identifying patient-ventilator asynchrony, as shown by another study, further support the significance of DL techniques in clinical forecasting. Additionally, the successful application of CNN by Hayasaka et al.^22^, in classifying intubation difficulties suggests its potential utility in emergency scenarios or under general anesthesia. Other study^23^ found that a 2-layer LSTM network outperformed rule-based algorithms and other machine learning models, achieving F1 scores of 0.983 and 0.979 for double triggering (DT) and inspiratory effort during expiration (IEE) detection, respectively, across datasets. It also surpassed alternative methods in cross-testing. These findings underscore LSTM's efficacy in accurately identifying patient-ventilator asynchrony (PVA) in clinical settings, potentially improving patient ventilator interaction by better detecting and correcting PVA^23^. Other studies^24,25^ have demonstrated that CNNs can effectively predict diseases and assist doctors in making better treatment decisions. Collectively, these findings emphasize the valuable role of DL models, particularly LSTM and CNN, in improving clinical decision-making and enhancing patient care.

Additionally, alternative findings from our study revealed that RF and XGB excelled among machine learning models. RF achieved high accuracy and specificity, while XGB demonstrated excellent sensitivity, F1-score, and ROC performance. These findings shed light on the potential utility of machine learning techniques, particularly RF and XGB, in clinical decision-making scenarios related to intubation necessity in methanol-poisoned patients. The high accuracy and specificity of RF suggest its suitability for precise identification of patients requiring intubation, which is crucial for timely intervention and improved patient outcomes. Conversely, the excellent sensitivity, F1-score, and ROC performance of XGB indicate its effectiveness in accurately capturing subtle patterns in the data, facilitating the detection of both intubation and non-intubation cases with high reliability. Siu et al.^5^, predicted intubation necessity within 24 h of critical care admission using standard bedside and lab parameters. Their study found that the random forest model achieved an AUC of 0.86, surpassing logistic regression. The random forest model demonstrated a sensitivity of 0.88 and specificity of 0.66, accurately predicting intubation while maintaining good calibration across various risk levels. These results suggest that machine learning, especially random forest, can effectively anticipate intubation needs in critically ill patients using routinely collected clinical data, potentially aiding real-time clinical decision-making shortly after ICU admission^5^. Arvind et al.^26^, predicted neonatal mortality during mechanical intubation for respiratory failure, they found that RF, bagged CART, and SVM models outperformed other algorithms. Additionally, García-García's.^27^, study demonstrated that our XGB pipeline was the most effective method for tracheal intubation, achieving the lowest number of false negatives at the optimal Bayesian decision threshold. Ding et al.^28^, also demonstrated that the XGBoost model, among various predictive models, yielded the highest AUROC (0.8353) for forecasting emergency Department intubation risk. The findings from this and our studies support the efficacy of machine learning techniques, with RF showing promise in anticipating intubation needs in critically ill patients, and XGB being the method of choice for tracheal intubation prediction in emergency department scenarios. These collective results underscore the potential of machine learning in enhancing clinical decision-making and patient care in critical situations.

Furthermore, our study revealed that ML algorithms consistently outperformed DL across all evaluation metrics, including Accuracy, Sensitivity, specificity, F1-score, and ROC. The superior performance of ML algorithms could be their ability to handle small or imbalanced datasets more effectively. In medical research, including our study on methanol-poisoned patients, acquiring large and balanced datasets for training DL models can be challenging. ML algorithms, particularly ensemble methods like RF and gradient boosting machines like XGB, are known for their^29,30^, which may have contributed to their better performance in our study. Furthermore, the choice of features or input variables may also play a role in the comparative performance of ML and DL models^31^. ML algorithms are often more flexible and adaptable to different types of input data and feature representations, allowing researchers to tailor the model inputs to the specific characteristics of the clinical dataset^32^. In contrast, DL models may require extensive feature engineering or preprocessing steps to effectively extract relevant patterns from the data, which can introduce additional complexity and potential sources of error. Overall, our study underscores the advantages of ML algorithms over DL models in predicting the need for intubation in methanol-poisoned patients. The interpretability, robustness to data characteristics, and flexibility in feature representation offered by ML algorithms make them well-suited for clinical decision-making tasks in medical contexts where transparency and performance are paramount. However, further research is needed to explore the potential benefits of integrating explainable artificial intelligence techniques into ML and DL models to enhance their interpretability and trustworthiness in real-world clinical applications.

Additionally, our study findings highlighted key predictors for intubation necessity in methanol-poisoned patients, including GCS at admission, ICU admission, age, and biochemical markers. The identified predictors for intubation necessity in methanol-poisoned patients, as highlighted in our study and supported by findings from Rahimi et al.^12^, shed light on crucial factors influencing patient outcomes in this specific clinical scenario. Among these predictors, GCS at admission emerges as a fundamental indicator of neurological status upon presentation. A lower GCS score suggests a more severe impairment of consciousness, indicating the potential need for airway management and mechanical ventilation to ensure adequate oxygenation and ventilation in patients with methanol poisoning, where central nervous system depression is a hallmark feature. ICU admission also emerges as a significant predictor, underscoring the critical nature of methanol poisoning and the likelihood of developing complications requiring intensive care support, including respiratory failure necessitating intubation^33^. The necessity for ICU admission reflects the severity of the poisoning and the need for close monitoring and intervention, including the provision of intubation if respiratory compromise ensues^34^. Age plays a notable role as a predictor, with younger patients exhibiting a higher likelihood of requiring intubation^35^. This finding may be attributed to various factors, including differences in metabolism, toxin clearance rates, and physiological resilience, which can influence the severity and progression of methanol poisoning. Younger individuals may also engage in riskier behaviors leading to higher methanol ingestion, further exacerbating the severity of poisoning and increasing the likelihood of respiratory compromise necessitating intubation.

Biochemical markers, such as elevated CPK levels, serve as indicators of tissue injury and metabolic derangement associated with methanol toxicity. Elevated CPK levels may signify multi-organ involvement and the extent of tissue damage, including myocardial injury, which can contribute to respiratory compromise and the need for intubation^36^. Additionally, specific visual symptoms associated with methanol poisoning, such as blurred vision or visual disturbances, may indicate ocular toxicity and central nervous system involvement, further reinforcing the need for airway management and supportive care, including intubation when indicated^36^. Overall, the identified predictors provide valuable insights into the clinical factors influencing the need for intubation in methanol-poisoned patients, informing risk stratification and guiding clinical decision-making to optimize patient care and outcomes in this challenging medical emergency. Integrating these predictors into predictive models leveraging explainable artificial intelligence techniques can enhance prognostication and aid clinicians in timely intervention and resource allocation, ultimately improving patient outcomes in cases of methanol poisoning.

### Study limitations

Due to the difficulties involved in gathering data from multiple hospitals across Iran, our research solely utilized data from a single hospital. To improve the applicability of results, forthcoming studies should contemplate enlarging the sample size or incorporating data from various provinces. Additionally, our study employed a limited set of eight models, comprising four DL and four ML techniques. To achieve a deeper insight, it is recommended for future research to investigate a wider range of models.

## Conclusion

Our study demonstrates the effectiveness of Explainable Artificial Intelligence (XAI) in predicting the necessity for intubation in methanol-poisoned patients. By comparing DL and ML models, we found that both techniques offer valuable insights, with ML models such as Random Forest (RF) and XGBoost (XGB) showing particularly promising results. ML models exhibited superior performance across various metrics, indicating their potential in clinical decision-making. Additionally, key features identified for predicting intubation necessity provide clinicians with valuable predictive indicators, including Glasgow Coma Scale (GCS) at admission, ICU admission, age, and biochemical markers. Overall, our study underscores the importance of leveraging XAI techniques to enhance predictive accuracy and aid in timely interventions for better patient outcomes in methanol poisoning cases. Moving forward, leveraging XAI techniques can enhance predictive accuracy and facilitate improved patient outcomes in methanol poisoning cases.

### Supplementary Information

Supplementary Information.

## Data Availability

The datasets generated and/or analyzed are not publicly available owing to ethical and legal causes. Nevertheless, they can be made available from the corresponding author Sayed Masoud Hosseini upon reasonable request.

## Abbreviations

AI: Artificial intelligence
ML: Machine learning
DL: Deep learning
XAI: Explainable Artificial Intelligence
CH3OH: Methanol
MDNN: Multimodal deep neural network
sCr: Blood glucose, serum creatinine
ALT: Alanine transaminase
CPK: Creatine phosphokinase
AST: Aspartate transaminase
ALP: Sodium, potassium, alkaline phosphatase
BUN: Blood urea nitrogen
SULOV: Uncorrelated List of Variables
MIS: Mutual Information Score
DNN: Deep Neural Network
FNN: Feedforward neural network
LSTM: Long Short-Term Memor
CNN: Convolutional Neural Network
XGB: Extreme Gradient Boosting
SVM: Support Vector Machine
DT: Decision Tree
RF: Random Forest
SHAP: Shapley Additive Explanations
LIME: Local Interpretable Model-agnostic Explanations
TP: True positive
FP: False positive
TN: True negative
FN: False negative
